# Resonance control of acoustic focusing systems through an environmental reference table and impedance spectroscopy

**DOI:** 10.1371/journal.pone.0207532

**Published:** 2018-11-14

**Authors:** Daniel M. Kalb, Robert J. Olson, Heidi M. Sosik, Travis A. Woods, Steven W. Graves

**Affiliations:** 1 Center for Biomedical Engineering, University of New Mexico, Albuquerque, New Mexico, United States; 2 Biology Department, Woods Hole Oceanographic Institution, Woods Hole, MA, United States; 3 University of New Mexico Center for Molecular Discovery, 1 University of New Mexico, Albuquerque, NM, United States; Helmholtz-Zentrum Dresden-Rossendorf, GERMANY

## Abstract

Acoustic standing waves can precisely focus flowing particles or cells into tightly positioned streams for interrogation or downstream separations. The efficiency of an acoustic standing wave device is dependent upon operating at a resonance frequency. Small changes in a system’s temperature and sample salinity can shift the device’s resonance condition, leading to poor focusing. Practical implementation of an acoustic standing wave system requires an automated resonance control system to adjust the standing wave frequency in response to environmental changes. Here we have developed a rigorous approach for quantifying the optimal acoustic focusing frequency at any given environmental condition. We have demonstrated our approach across a wide range of temperature and salinity conditions to provide a robust characterization of how the optimal acoustic focusing resonance frequency shifts across these conditions. To generalize these results, two microfluidic bulk acoustic standing wave systems (a steel capillary and an etched silicon wafer) were examined. Models of these temperature and salinity effects suggest that it is the speed of sound within the liquid sample that dominates the resonance frequency shift. Using these results, a simple reference table can be generated to predict the optimal resonance condition as a function of temperature and salinity. Additionally, we show that there is a local impedance minimum associated with the optimal system resonance. The integration of the environmental results for coarse frequency tuning followed by a local impedance characterization for fine frequency adjustments, yields a highly accurate method of resonance control. Such an approach works across a wide range of environmental conditions, is easy to automate, and could have a significant impact across a wide range of microfluidic acoustic standing wave systems.

## Introduction

Acoustic standing waves enable rapid and precise focusing of biological particles and cells within a flowing fluid sample. When paired with either precision optical analysis or microfluidics, the uses of acoustic standing wave devices are numerous and include isolation of particles and cells based on size and compressibility differences [1], enrichment of rare cells of interest (e.g. circulating tumor cells) from blood [2–4], centrifuge- free washing of cells from sample media [1], as well as focusing for field-based interrogations such as electrical impedance spectroscopy [5] or flow cytometry [6–9]. Within the field of flow cytometry, acoustic focusing can be particularly valuable for systems that have inherently dilute particle concentrations such as environmental monitoring of microalgae and diatom populations. For example, the integration of in-line acoustic focusing into Woods Hole’s Imaging FlowCytobot has been demonstrated to drastically increase the volumetric throughput and event rates through the cytometer (up to 10 fold), without sacrificing optical performance.[9]

Acoustic standing waves are typically generated using either bulk acoustic waves (BAW) or surface acoustic waves (SAW). Although the fundamental equation governing the primary acoustic force on the particles remains the same for both BAW and SAW waves[10], there are distinct advantages and drawbacks to each of these excitation methods. The excitation of the SAW is generally achieved by driving two sets of interdigital electrodes on opposite sides of the microfluidic channel, while some recent work has demonstrated SAW systems capable of creating focusing nodes with a single set of interdigital electrodes[11, 12]. Both of these methods for driving a SAW system yield a highly compact platform on a single substrate with channels that can be fabricated from less rigid materials (e.g. Polydimethylsiloxane (PDMS)). By contrast, BAW systems are simpler to construct (they can be as simple as a capillary made from glass or steel, or an etched channel in a silicon wafer)[6, 13] and have demonstrated significantly higher volumetric throughputs[13].

In BAW devices, the standing wave is generated by a piezoelectric drive, most commonly a ceramic Lead Zirconate Titanate (PZT) transducer, physically bonded to the substrate. BAW devices are typically excited at the appropriate drive frequency to create an integer numbers of half wavelengths across the width of the microfabricated channel or capillary cross section. Although wall materials, coupling layers, and other factors have an effect, the drive frequency of a device is largely determined by the channel dimensions along with the speed of sound within the sample[14]. As the speed of sound in an aqueous solution is dependent on both the temperature and salinity of the solution, functional BAW devices must either regulate temperature and salinity or actively control the drive frequency to account for changes in the speed of sound in the system. Precise temperature and media control has been successfully demonstrated as a methodology of maintaining consistent acoustic performance over time for both system characterization[15] and as a resonance control strategy[16, 17]. Despite its effectiveness, precisely controlling temperature and salinity of the media may not be an effective strategy for a multi-user instrument, resource poor areas, or fieldwork without highly controlled environmental conditions.

Although a trained operator can actively shift the driving frequencies to maintain high focusing performance, such an approach is not subject to automation and can lead to inconsistent performance over time. Particularly challenging application spaces for manual resonance control include remote flow cytometry for analysis of oceanic samples[18, 19], routine instrument use by untrained personnel, and fully automated separation systems for large scale enrichment of rare cells. For this reason, we have developed a robust method of resonance control to select and maintain the ideal operating frequency for BAW systems across a wide range of sample and environmental conditions. Our approach, described in detail below, is simple, low cost, and easy to automate, making it amenable to numerous applications. We have demonstrated this approach in both capillary and etched silicon devices to show the potential value of this method across a variety of instrumentation platforms.

## Materials and methods

### Acoustic focusing devices and optical analysis

Two acoustic focusing devices were built and tested for the resonance control experiments. The stainless-steel capillary was used for its ease of manufacturing, high performance, and inherent symmetry that allows for the driving of a 2D focusing mode for flow cytometric analysis of particles[7, 8]. The etched-through silicon wafer platform, which is highly customizable and well-suited for precise particle separations, was tested to confirm and generalize the capillary results.

#### Steel capillary

**Fig 1:**
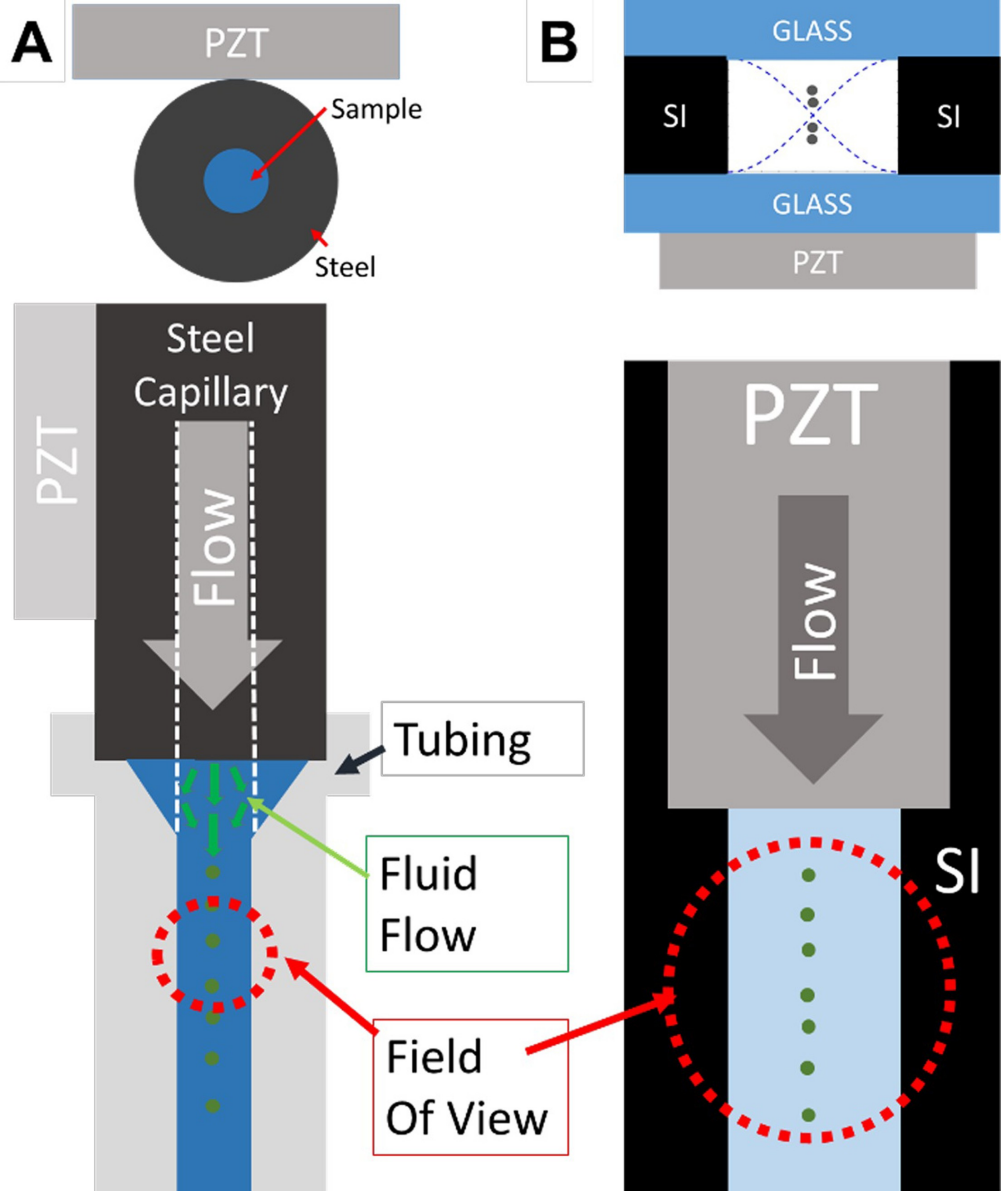
Schematic of the two acoustic focusing devices and their optical analysis points. Within both of these systems, the one-dimensional focusing of particles was monitored downstream of the driving PZT. Both of these systems were driven at their fundamental half-wavelength mode, represented by the blue dashed lines in B top, which focused the particles into a single central node. The focusing performance of the capillary (A) was monitored through transparent tubing at the end of the device while the etched-through Si wafer (B) is monitored just downstream of the PZT.

A stainless-steel capillary (1/16” OD X 500 μm ID x 20 cm in length) (Vici Valco T20C20-10) was driven by a 1.5 MHz thickness mode PZT (5 mm width x 30 mm length) directly bonded to the capillary using a fast-curing cyanoacrylate superglue. Although the image in Fig 1 represents the superglue bonding the PZT to the steel as a pure line, in practice we observed that it forms a wetted meniscus that effectively increases the surface area in contact with the steel capillary. It is possible that rigid mounting or clamping of the steel capillary, especially near the driving PZT, can shift acoustic performance, to eliminate this effect we tend to use non-rigid mounts at the end of the capillary. Beyond its ability to drive a high performance 2D focusing mode, the stainless-steel capillary was used for the relative ease of integrating it into an imaging flow cytometer that monitors phytoplankton in remote oceanic environments [9, 18]. Within the flow cytometer, the capillary directly replaced the cytometer’s sample delivery needle into the optical flow cell without any major system modifications. The tight acoustic focusing of the sample prior to analysis allowed the system to increase the volumetric flow rate of the sample without increasing the linear velocity of the particles though the system.

**Fig 2:**
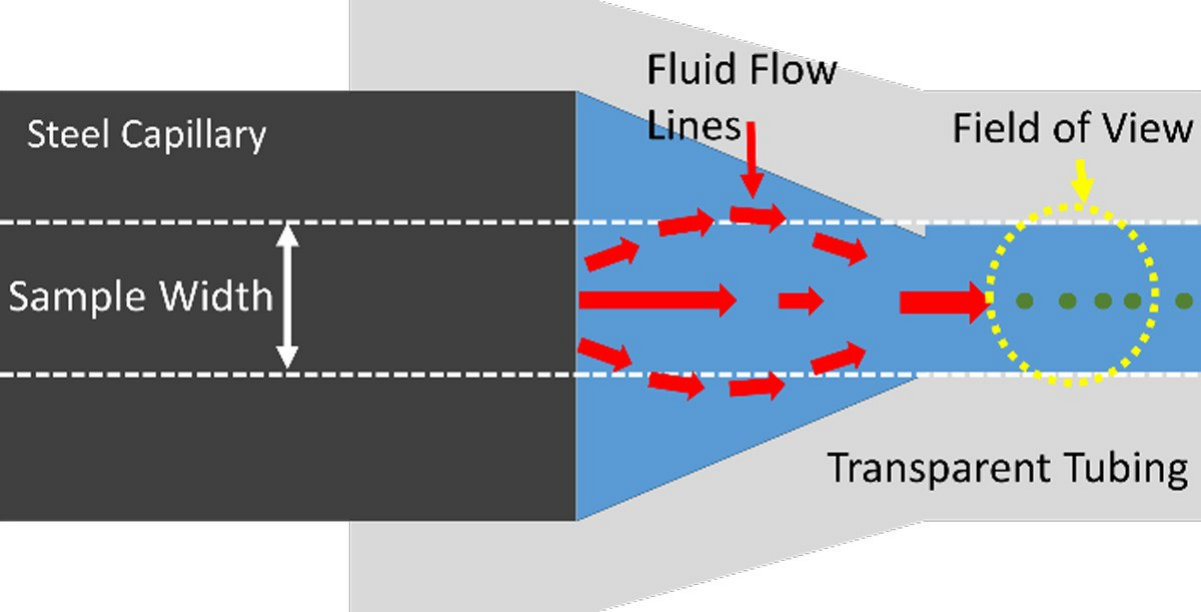
Close-up of capillary analysis technique. Imaging was done through a simple, flexible tubing to continuously monitor the particle positions after they exit the steel capillary.

The focusing position of the particles within the stainless-steel capillary was monitored with an epifluorescence microscope and a high-speed camera (Andor Luca S, Belfast, Ireland) in a temperature controlled room. The focusing width at the downstream end of the capillary was continuously monitored through transparent tubing. Notably, for our excitation and emission points, we achieved high quality fluorescent imaging through this tubing (Tygon R3607, ID 1.02 mm, Wall 0.86 mm, IDEX Health & Science, Oak Harbor, WA). This development may have the potential to be used as an inexpensive and simple imaging or cytometry platform (Fig 2).

#### Etched-through Si wafer

Using a microfabrication technique similar to that described by Suthanthiraraj et al.[20], a Si wafer was deep reactive-ion etched (DRIE) through the entire wafer thickness (500 μm) to a width of ~600 μm and a length of ~5 cm. Glass slides were anodically bonded to both sides to create a transparent device. A 1.5 MHz PZT was bonded directly under the channel and driven at the fundamental half-wavelength frequency across the channel width (~600 μm, ~1.2MHz) to create a single focusing node. The etched-through Si wafer was imaged just downstream of the focusing PZT utilizing a custom built flow cytometry instrumentation platform that has previously been described.[21] In short, this platform (S1 Fig) uses a line focused laser (600 μm across device width, ~20 μm in direction of flow), wide-field collection optics, and a high-speed camera (Hamamatsu, Bridgewater, NJ) to extract the position of the focused parties across the width of the Si device.

### Quantification of acoustic focusing

The 1D width of focusing within an acoustic focusing device is a function of a variety of factors including the flow rate, the particle and media characteristics, the input acoustic power, and the applied excitation frequency. Holding the other variables constant, examining how the focusing width varies with the applied acoustic frequency yields an experimental method to determine the optimal resonance frequencies (where the tightest particle focusing occurs). It is desirable to run at this high-performance condition for both analysis and separation applications.

**Fig 3:**
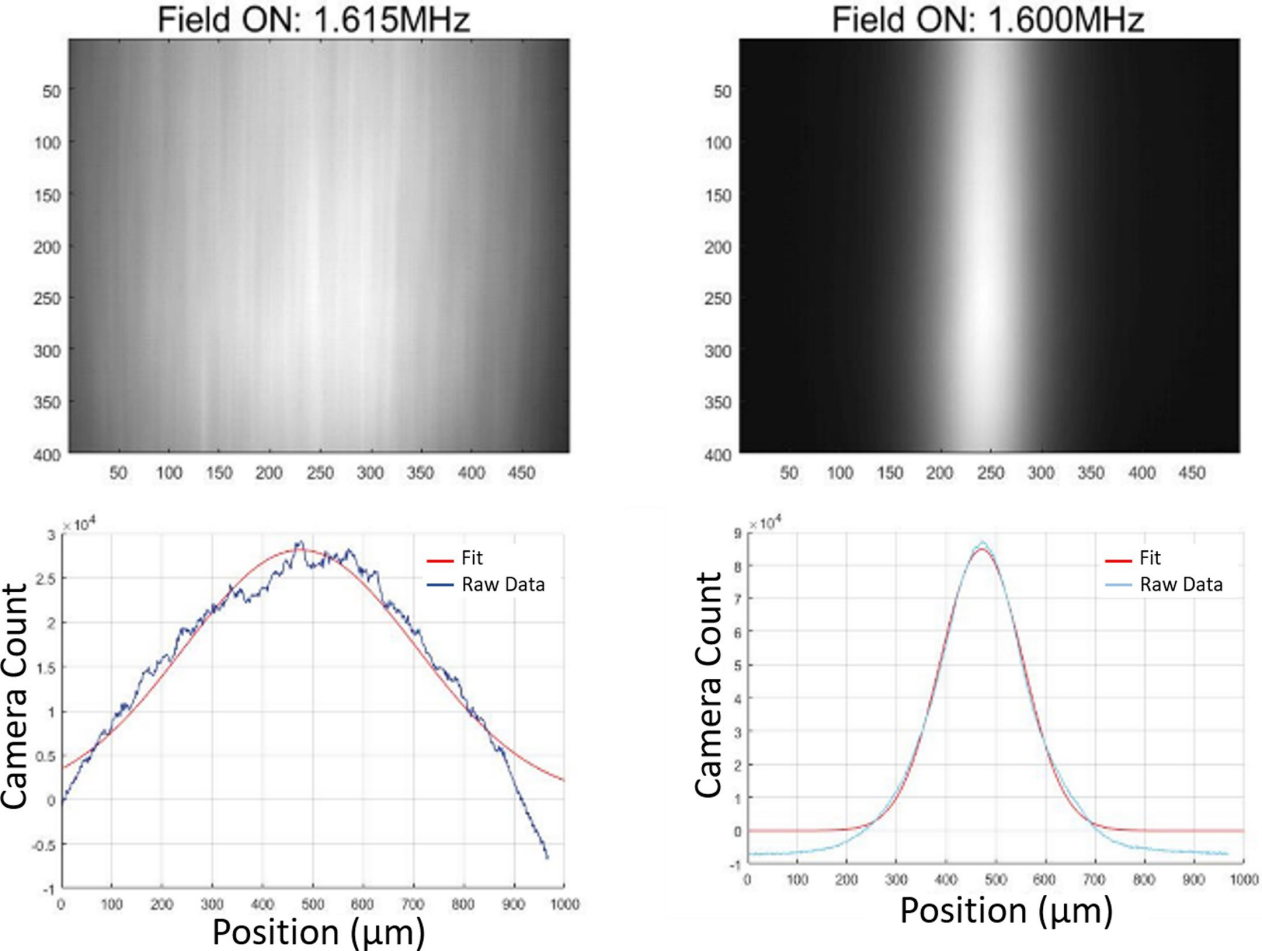
Quantification of focusing. For a model system of fluorescent 10- μm beads (Sky Blue FP-10070-2, Spherotech), an example of poorly focused (left) and well-focused image data (right). Each image shown here was constructed from a summed stack of 500 individual images taken over ~30s of data at a single frequency condition. The focusing width of each summed image was fit to a Gaussian curve using MATLAB and the width at half-maximum was used as a representative focusing width. Applying this process across an iterative frequency scan for a single temperature yields an experimental method of measuring the optimal resonance frequency condition. Repeating this process at a variety of temperatures allowed for the experimental characterisation the highest performing resonance frequencies as a function of temperature.

Using the image data from our optical systems, we tracked the positon of the particles over time and were able to quantify the particle focusing width across any range of conditions (e.g., Fig 3).

### Temperature control and monitoring

The focusing performance of the steel capillary device was monitored with a fluorescent microscope (Fig 1) in a temperature-controlled room. A consistent model sample, 10-μm polystyrene micro-particles (Sky Blue FP-10070-2, Spherotech, Lake Forest, IL), was used to characterize the focusing performance across a broad test matrix of temperatures and frequencies. The thermal conductivity of the steel acoustic focusing system to the surrounding room made it necessary to subject the entire system, not just the input sample, to a variable temperature environment. The entire system, including the input sample and device temperature, was allowed to fully equilibrate (at least one hour) to the ambient condition prior to each test. The ambient temperature, the capillary temperature, the input liquid temperature, and the output liquid temperature were all continuously monitored throughout the experiments. Due to heating of the driving PZT, typical results show that the liquid sample experiences a small temperature increase, ~2C over the ambient and sample input temperatures, when measured at the end of the acoustic focusing system.

### Salinity experiments

Distilled (DI) water and f/2 media [22] were mixed at appropriate concentrations to create five media standards with salinities ranging between 0–30 g/L. Ten-μm fluorescent polystyrene particles (Sky Blue FP-10070-2, Spherotech, Lake Forest, IL) were suspended in the five media standards and the focusing performance was monitored across a wide range of acoustic excitation frequencies. All salinity experiments were conducted at room temperature (23°C) with continuous temperature monitoring. The salinity experiments were conducted within both the capillary and etched silica systems and the focusing performance was monitored across a wide frequency space at each salinity condition.

### Automation, impedance signal acquisition, and calibration

**Fig 4:**
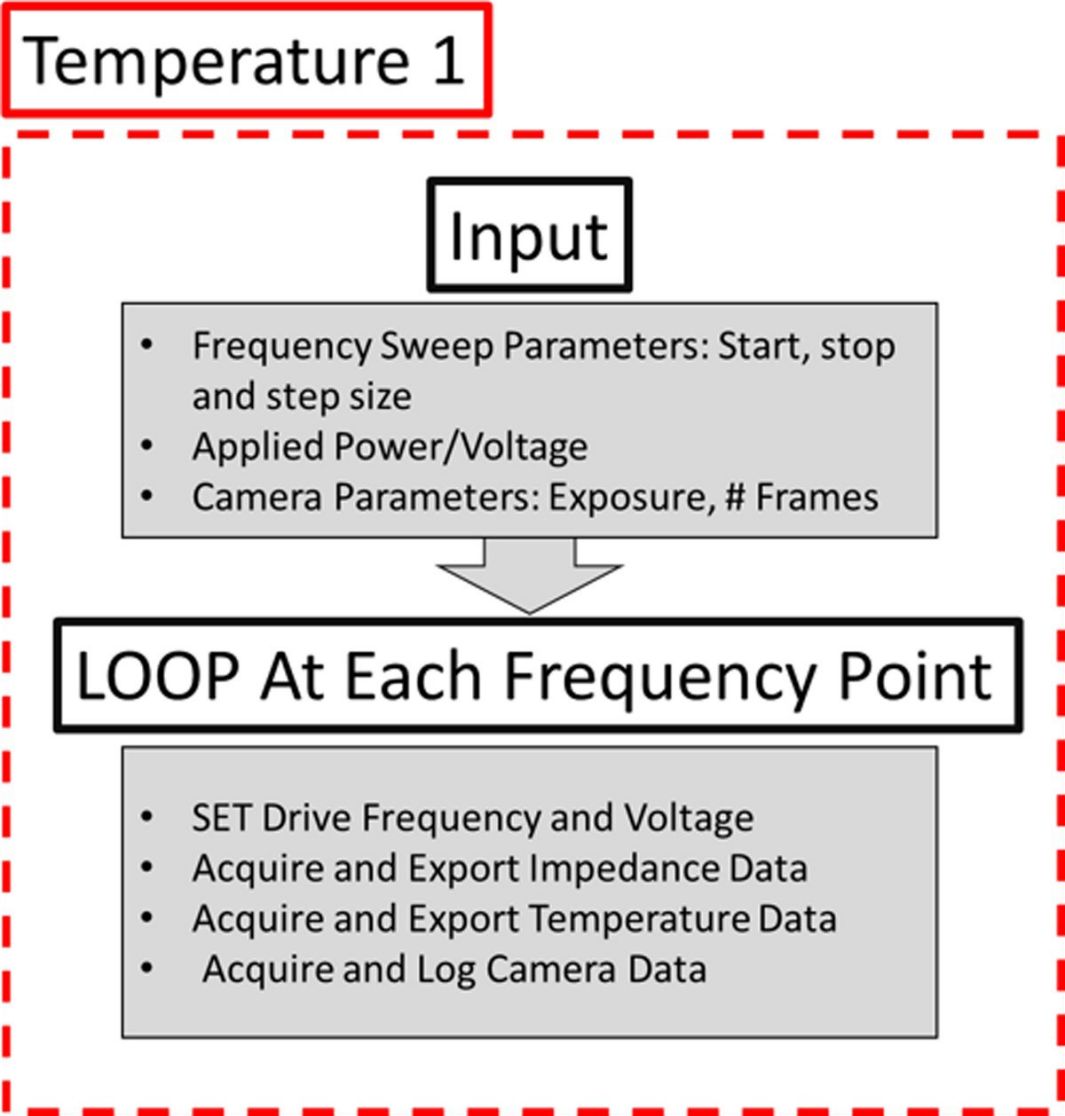
Control logic used for the frequency scans at each discrete temperature point. This automated scan allows for the collection and analysis of the system’s frequency-dependant focusing performance at any particular temperature point.

The acoustic frequency sweeps and all of the data acquisition was fully automated through the use of LabVIEW. At every single driving frequency of interest, the camera image data, the acoustic impedance information, and the temperature data were continuously logged and exported. Typically, the frequency search bandwidth was at least 50 kHz with 1 kHz step sizes. Large manual scans were done beyond these frequency points to confirm that there were no other frequency regions that exhibit focusing. We note that while the impedance scans *could* be acquired very rapidly (~1 ms per frequency point, ~50 ms for full scan across 50 kHz) our need to acquire large number of images to accurately quantify particle focusing yielded much longer scan times (~30 s of 500 images at each frequency point, ~25 minutes per full 50 kHz scan). The image information was analysed offline in MATLAB as described above, while the four temperature probes (input sample temperature, output sample temperature, ambient temperature, and device temperature) were logged at every frequency point of interest. This process (Fig 4) was repeated across seven discrete ambient temperatures points from 8°C to 37°C.

The desire to simultaneously drive the system and collect the impedance information without any downtime led us to utilize a function generator and oscilloscope, instead of an external impedance analyser, to acquire the impedance information. Our impedance information was acquired by logging the voltage (measured in parallel with the PZT), current (Pearson current Loop), and relative phase information from the driving PZT at every frequency point. To ensure that our impedance information was accurate, an Agilent impedance analyser (Santa Clara, CA) was used as a ‘gold standard’ on a standard (10Ω resistor). This standard was then cross-calibrated to our LabVIEW/Function Generator/Oscilloscope system across the desired frequency space. The necessary corrections to the complex impedance data (real and imaginary parts) were then adjusted such that our system agreed with the Agilent as a ‘gold standard’ (S2 Fig).

### COMSOL model

The acoustic module of COMSOL Multiphysics was used to model the 1D resonance response of the PZT, steel capillary, and liquid sample system. For a variety of system temperatures, an eigenfrequency scan around the predicted frequency of ~1.5 MHz was conducted and the frequency of the fundamental half-wavelength eigen-mode, the condition where the acoustic focusing energy is maximized, was selected as the optimal frequency for that particular temperature (S3 Fig).

### Speed of sound model

In addition to the COMSOL model, a simple model was built in-house to predict how the resonant frequency will shift with temperature and salinity changes. This model assumed that all resonance frequency shifts are determined from changes in the media’s speed of sound alone, it did not take into account any other system effects. Beginning with a simple wave equation (c=fλ), the resonance frequency (f) required to maintain the half-wavelength resonance at a constant system length (λ/2), can be expressed as a function of speed of sound (c).

$$\text{f}\left(\text{c}\right)=\frac{2c\;\left(T,S,D\right)}{\lambda }$$ (1)

The speed of sound in water is a function of the temperature in °C (T), salinity in g/L (S), and water depth (D). It can be calculated at any environmental condition using the Mackenzie equation (Eq 2).

$$\text{c}\left(\text{T},\text{S},\text{D}\right)=1448.96+4.591\text{T}-5.304\text{*}10^{-2}{\text{T}}^{2}+2.374\text{*}10^{-4}{\text{T}}^{3}+1.34\left(\text{S}-35\right)+1.630\text{*}10^{-2}\text{D}+1.675\text{*}10^{-7}{\text{D}}^{2}-1.025\text{*}10^{-2}\text{T}\left(\text{S}-35\right)-7.139\text{*}10^{-13}{\text{TD}}^{3}$$ (2)

Substituting the Mackenzie equation (Eq 2) back into the wave equation (Eq 1), this simple model predicts the half-wavelength resonance frequency as a function of temperature, salinity, and water depth (which is zero in our model). For accurate prediction of resonance frequencies shifts, this model must first be related to a single known condition of optimal performance both in terms of frequency (f_reference_) and speed of sound based upon environmental conditions (c_reference_). Assuming a constant half-wavelength resonance length (λ/2), this method allows us to predict (Eq 3) what shift in resonance frequency is required to maintain maximal performance as the environmental conditions and speed of sound shift.

$${\text{f}}_{\text{predicted}}={\text{f}}_{\text{reference}}\frac{{\text{c}}_{\text{predicted}}}{{\text{c}}_{\text{reference}}}$$ (3)

## Results

### Acoustic resonance frequency selection and power selection for frequency scans

**Fig 5:**
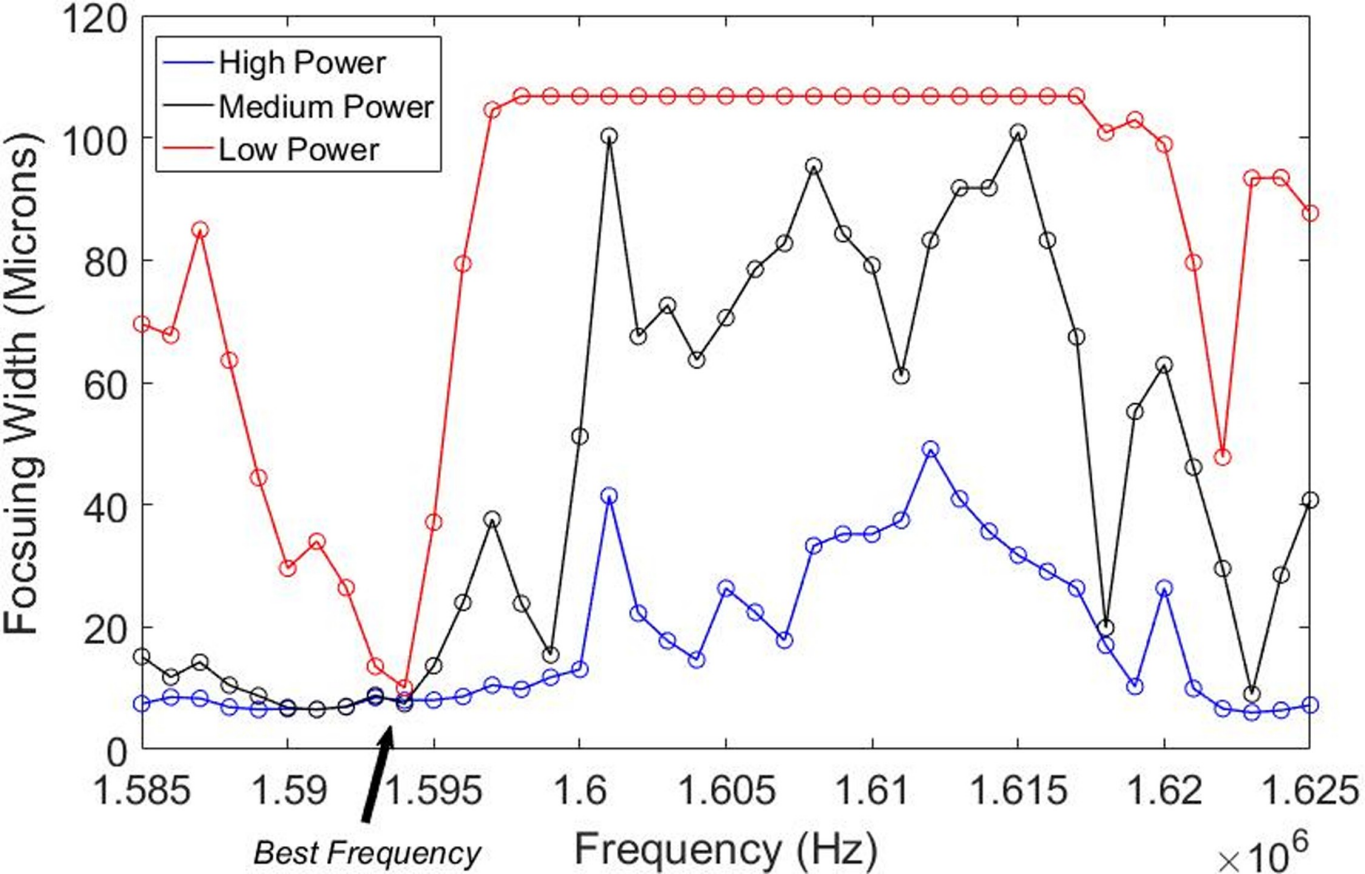
Focusing width vs frequency for three input power levels within the cylindrical capillary system. At the highest input power levels there was tight focusing across a wide frequency space, while at the lowest input power there was tight focusing (less than ~20 μm) across a small frequency bandwidth of a few kHz.

**Fig 6:**
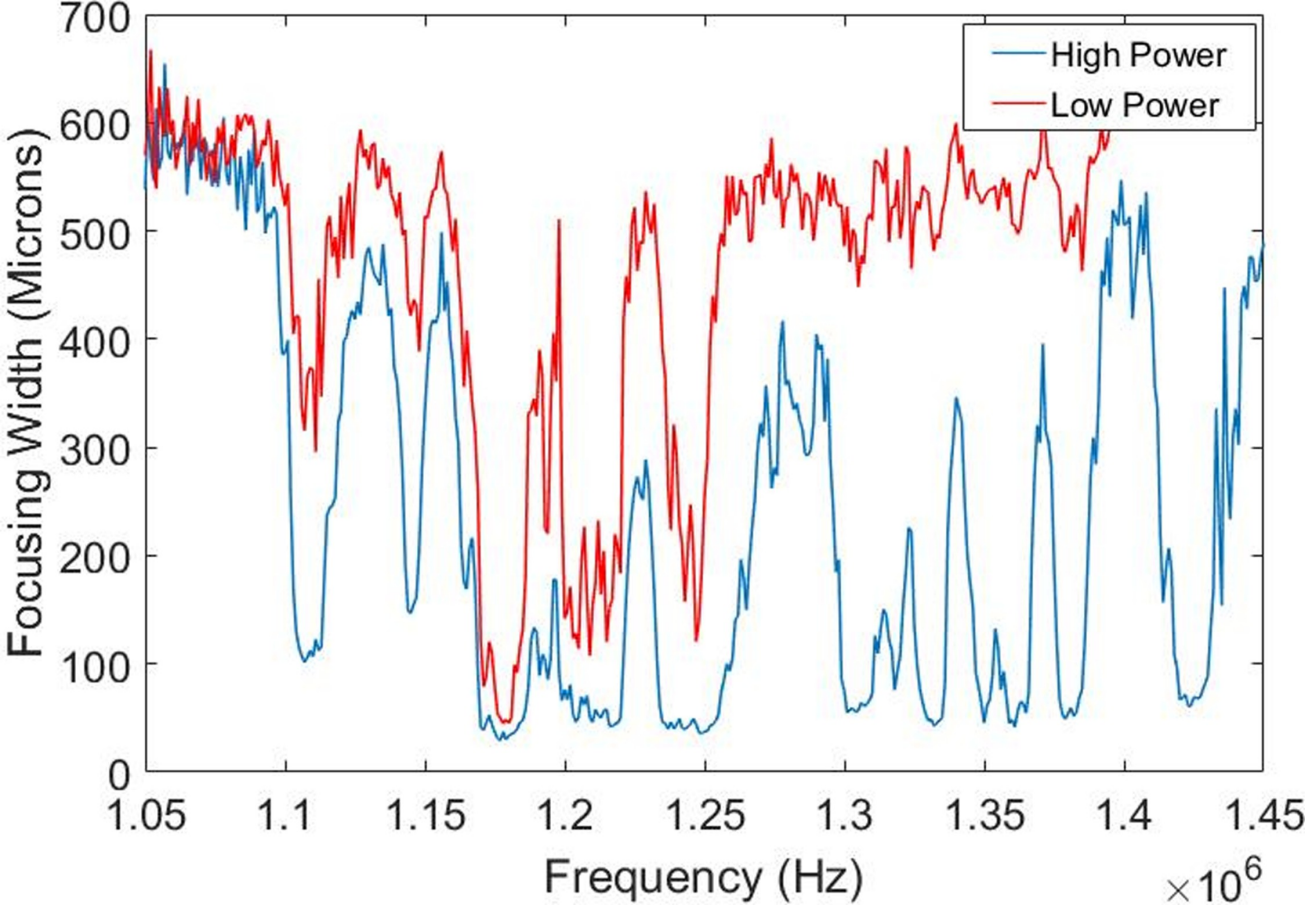
Power dependence to focusing width in etched silica device. For the Si etched device at low input power there was a single frequency bandwidth around 1.175 MHz that resulted in optimal focusing performance. At the high power level there were multiple local minima of high focusing performance.

The focusing performance of a resonant standing wave acoustic system is highly dependent on the excitation frequency. Using our methodology to quantify the focusing width, we were able to sweep across the relevant frequency space, acquire the time lapse images and select the appropriate frequencies where the focusing width of the particles is minimized. For the cylindrical capillary system, we observed tight focusing across a wide frequency space at the highest input power levels, but at the lowest input power, tight focusing (less than ~20 μm) only occurred across a small frequency bandwidth of a few kHz (Fig 5). For the Si etched device, we similarly found that a single frequency bandwidth (around 1.175 MHz) resulted in optimal focusing performance at low input power, while at the high power level there are multiple local minima with high focusing performance (Fig 6).

It is highly desirable to operate at and maintain the *best* resonance frequency of the system. While high input powers generally have large bandwidths and/or multiple minima of tight focusing, applying a low input power level generally limits focusing to a single narrow frequency bandwidth. Thus, exciting the system at low input powers allowed us to more accurately quantify the optimal frequency point(s) of interest.

### Temperature dependence of optimal focusing frequency

As described in the methods, the steel capillary system was exposed to a wide range of ambient and sample temperature conditions in a temperature-controlled room. Acoustic frequency sweeps monitoring the focusing, temperature, and impedance information were conducted at seven discrete ambient temperature points ranging from 8°C up to 37°C. Because driving the PZT produces heat, the device temperature (measured by a thermocouple attached to the steel capillary) and sample output temperature were a few degrees C warmer than the ambient temperature. The device and sample output temperatures were monitored, with the sample output temperature being selected as the characteristic temperature of the system.

**Fig 7:**
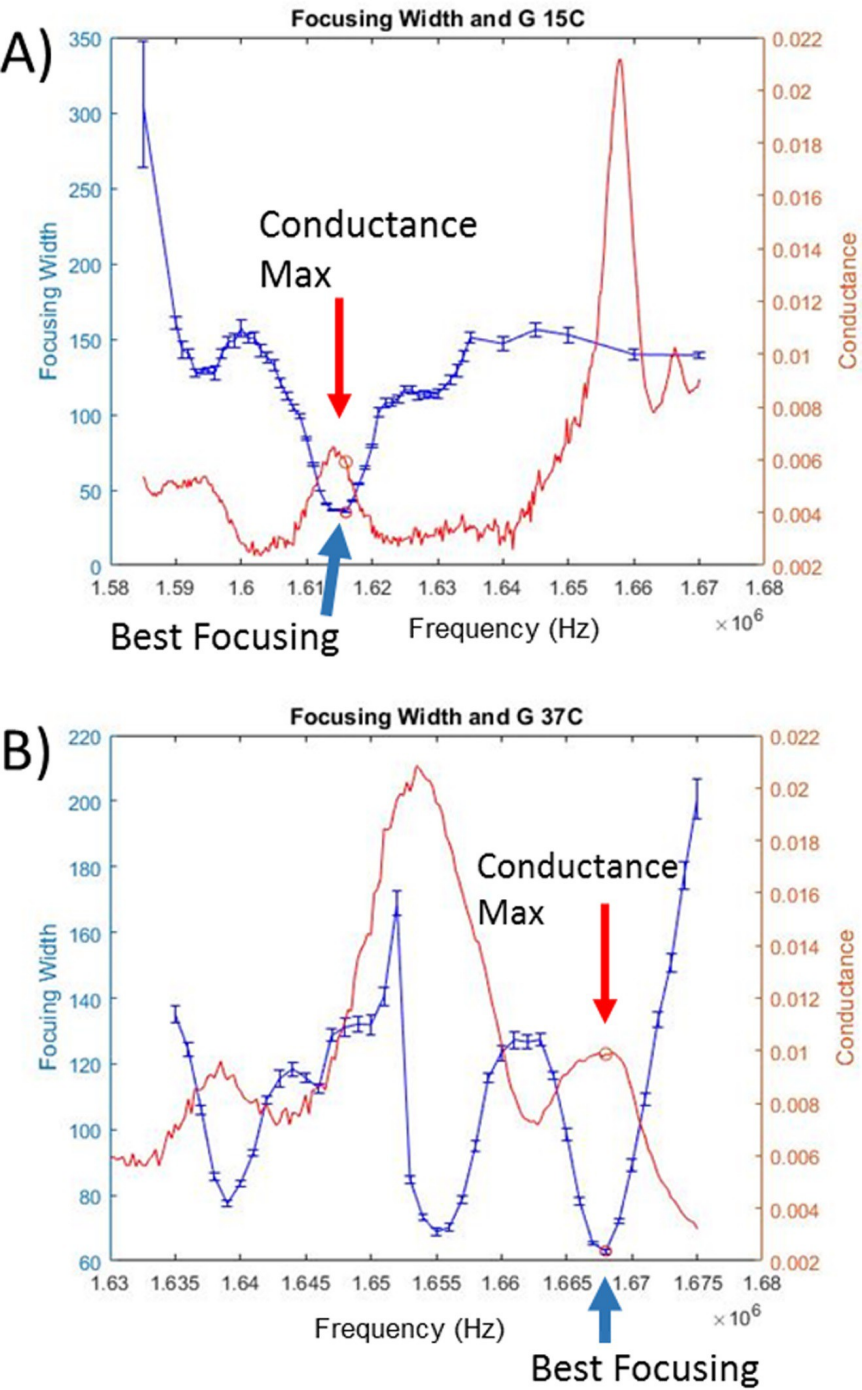
Capillary focusing and conductance spectra at two temperatures. Focusing width (blue) and conductance information (red) for two representative temperature spectra. A) Frequency scan, conducted at 15°C ambient, demonstrates one clear focusing minimum with significantly better focusing than any other frequency range. Additionally, this focusing minimum corresponds to a local conductance maxima of similar frequency bandwidth. Circles on each plot mark the highest performing frequency. B) Frequency scan, conducted at 37°C, has one global minimum where focusing performance (blue) is maximized, but also has two additional minima with high focusing performance. Again, there is a local conductance maximum (red) coinciding with the best operating frequency.

**Fig 8:**
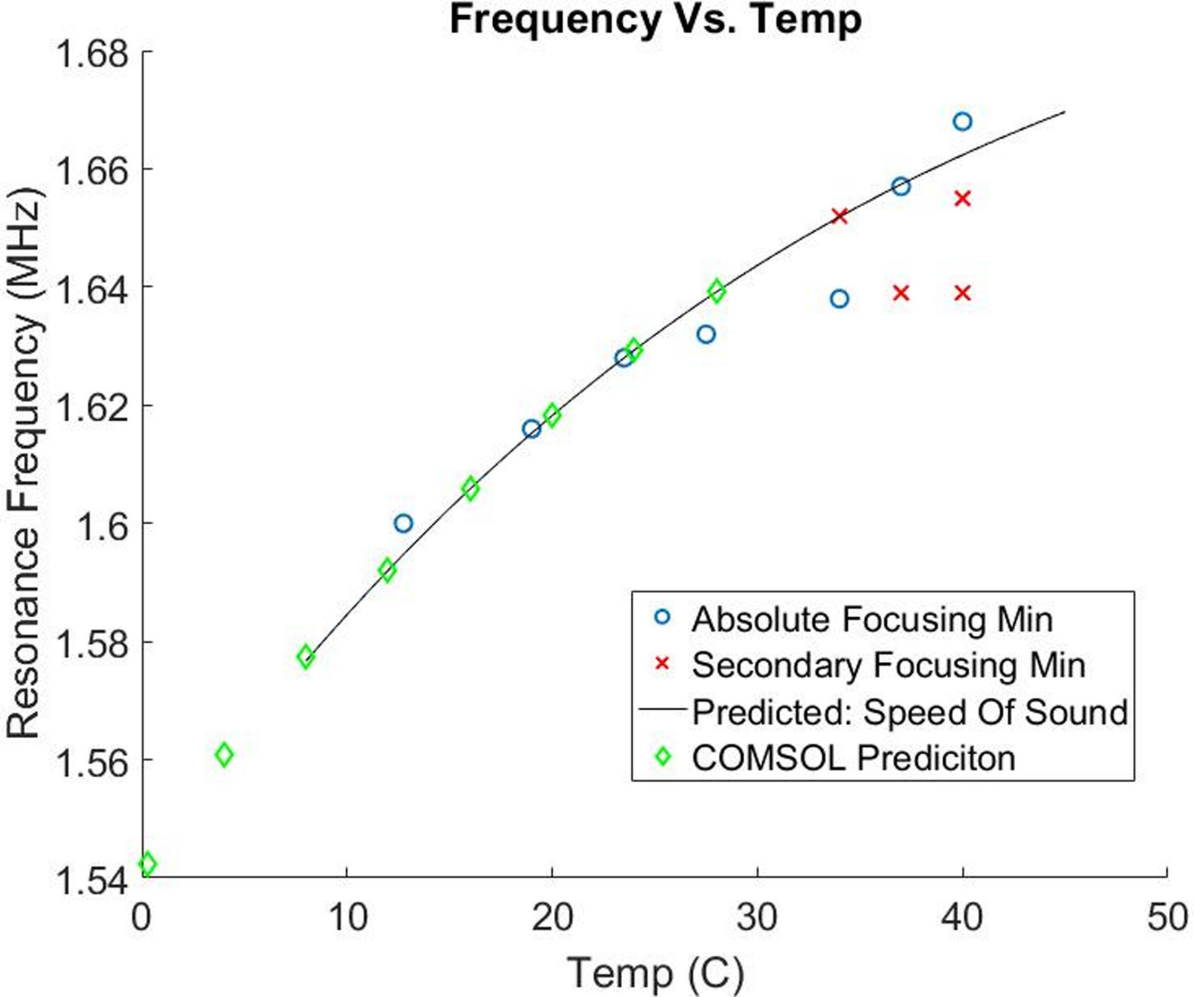
Capillary resonance frequency vs. temperature. Experimental data and two models characterizing how the resonance frequency of the system changed with temperature (*note the black line is a model, not a fit to the data*). The experimental data (blue for absolute focusing width minima, red for secondary focusing width minima) was well characterized by both models, suggesting that for this system, the speed of sound within the liquid layer dominates the resonance frequency shift.

A spectrum of the acoustic focusing width vs. frequency was obtained at every temperature point and the optimal operating frequency, defined as the point where the focusing width was tightest, was selected at each of these conditions. Most of these frequency sweeps have one clear minimum where the focusing width was minimized and the acoustic performance was highest. An example of such a spectra is shown in Fig 7 panel A where there is one clear focusing width minima across the frequency space. In contrast, some of the highest temperatures exhibited more complicated spectra with multiple minima of nearly equal performance (e.g. Fig 7B); in each of these cases the additional minima were also characterized as significant in the final data plot (Fig 8). Representative data plots showing both cases are shown below (Fig 7) and summary plots with all seven spectra are shown in the supporting information (S4 Fig).

#### Temperature dependence of optimal focusing frequency

After seven temperature points are fully analysed, the ideal resonance frequency was plotted as a function of temperature (Fig 8). At the highest temperatures with multiple minima, multiple frequency points were represented accordingly. In addition to this experimental data, two independent models were compiled and plotted. The 1D COMSOL model, as described in the methods, selected the fundamental half-wavelength resonance frequency at seven discrete temperature points. The second model assumes that temperature of the liquid layer alone determines the optimal resonance frequency of the capillary system (full description in methods).

The high correlation between these models and the experimental data, especially at lower temperatures, suggests that the speed of sound within the liquid layer dominates these frequency shifts with temperature.

### Salinity dependence of optimal focusing frequency

**Fig 9:**
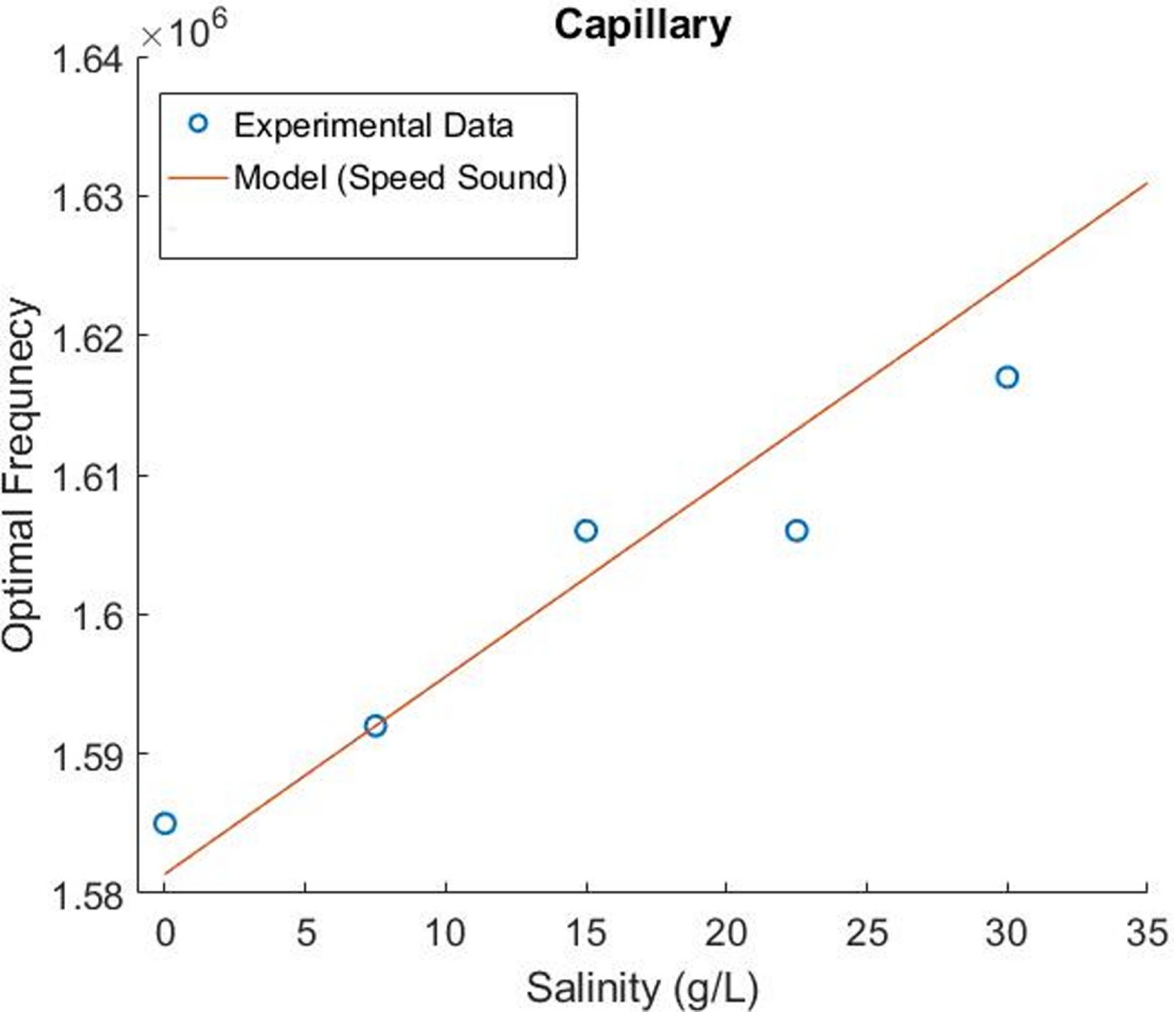
Optimal focusing frequency over a range of media salinities within the capillary system. The five blue scatter points were experimentally determined at each salinity while the red line plot represents a simple model that assumes that the speed of sound within the liquid media alone determines these resonance frequency shifts.

Much like the temperature experiments, the focusing performance of the acoustic focusing capillary system was characterized across a wide frequency space at five discrete media salinities. At each of these five media conditions, the frequency point where the focusing width was minimized was selected as the optimal resonance frequency. Fig 9 plots the highest performing frequencies vs. salinity at each of these five media conditions ranging from 0–32.5 g/L. The individual plots of the focusing width vs. frequency plots at each of these five media conditions, are shown in the supporting information (S5 Fig). As with the temperature data, the same model is used to predict resonance frequency shifts according to changes in media’s speed of sound.

Compared to the temperature experiments, we observed more discrepancies between the experimental data and the speed of sound model in the salinity case. However, the model does characterize the trend that increasing media salinity will increase the resonance frequency. These results will be more fully explored within our discussion where we show that both a simple lookup table and this model sufficiently limit the resonance frequency search bandwidth (e.g. such that the local conductance scan for resonance frequency operation would be within this search bandwidth).

In addition to the capillary system, the salinity effects were examined within the etched silica system. The results within the silica system (S6 Fig) showed a similar trend to the capillary system, where increasing salinity increases the optimal driving frequency. Despite this general similarity, the silica system does exhibit what appears to be a nearly binary shift between two local frequency points of high performance. In all likelihood, we are driving a more complicated system mode in this device, where increasing salinity shifts the best performing frequency from one local conductance peak to another.

### Local conductance max at resonance

Simultaneous logging of the focusing data and the impedance data allowed us to compare how the impedance parameters vary with focusing performance. For the capillary system, there was a local conductance maximum that correlates with the best focusing performance (Fig 7). We extended our studies to a DRIE etched-through Si wafer system to confirm that this observation holds within this popular instrumentation platform.

**Fig 10:**
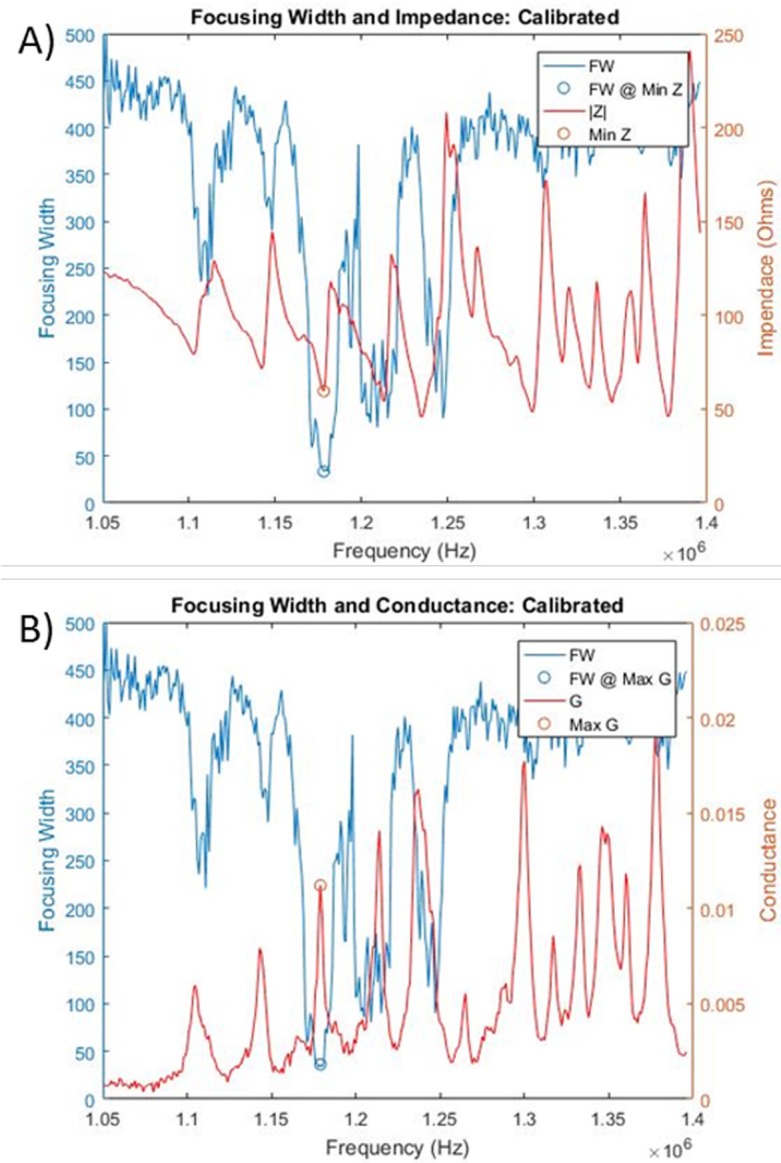
Focusing width and conductance in Si system. Calibrated impedance (A) and conductance (B) scans are shown overlaid with acoustic focusing width. A local conductance max (impedance minima) was correlated with the best performing driving frequency (1.179 MHz). The overlaid blue and red circles were selected from the focusing data alone, demonstrating near perfect correlation between the conductance maxima and focusing minima. Note that there were additional conductance peaks that could otherwise be indistinguishable from the optimal frequency if the search bandwidth was not limited.

Much like the capillary system, there was a local conductance maxima that was related to the optimal performing acoustic driving frequency in the Si wafer system (Fig 10). The bandwidth (frequency width at half max) of the conductance peak was 3.81 kHz, while the focusing bandwidth at this input power was 17 kHz (S7 Fig). Thus, in addition to being a much faster measurement, the smaller bandwidth of the conductance plot allows for more precise control of the frequency when compared to the image data alone.

This observation can be extended to high input powers that exhibit multiple local focusing minima. Much like the low power data, each of the highest performing frequencies are correlated to a local conductance maxima (S8 Fig).

## Discussion

We developed a robust methodology to determine the optimal resonance frequency for an acoustic standing wave system. Sweeping the excitation frequency across a wide band of frequencies, while simultaneously quantifying the focusing performance, allows for a simple yet robust characterization of the optimal resonance frequency across the frequency space. In our steel capillary-based system, our models suggest that these frequency shifts are dominated by the speed of sound within the liquid layer. Although we cannot yet generalize the assessment that the liquid speed of sound alone is sufficient to characterize frequency shifts, our coupled frequency scan with image analysis methodology could be used to characterize any particular system of interest. When combined with logging of the electrical parameters of interest (voltage, current, and phase) and changes in environmental temperature conditions, this technique allows for detailed characterization of the resonance conditions and allows for the development of a resonance control system.

Our experiments indicate that, over a wide range of temperature conditions, impedance spectroscopy alone cannot sufficiently characterize the ideal operating resonance frequency for an acoustic focusing system. Although there is a clear local conductance maximum associated with the tightest focusing resonance frequency condition, the spectra we have examined have other non-local maxima that cannot be distinguished from the optimal performing frequency unless the frequency search bandwidth is sufficiently limited (see Fig 10). This search bandwidth can be limited by rigorous experimental control of the system, such as maintaining both the sample and the acoustic focusing device at consistent temperatures (less than few °C variability), or can be accounted for by characterizing how these frequencies shift with temperature and salinity.

Our experimental characterization of how the resonance frequency shifts with temperature and salinity demonstrates how a simple reference table could be generated to predict the resonance within a few kHz bandwidth. Within this table, one could interpolate between any number of experimentally observed conditions by modelling the frequency shifts as being dominated by the speed of sound within the liquid layer. Although there is some scatter between the speed of sound model and the experimental characterization, this environmental condition only needs to be within a ~10 kHz bandwidth to successfully limit an impedance search to the correct frequency region.

**Fig 11:**
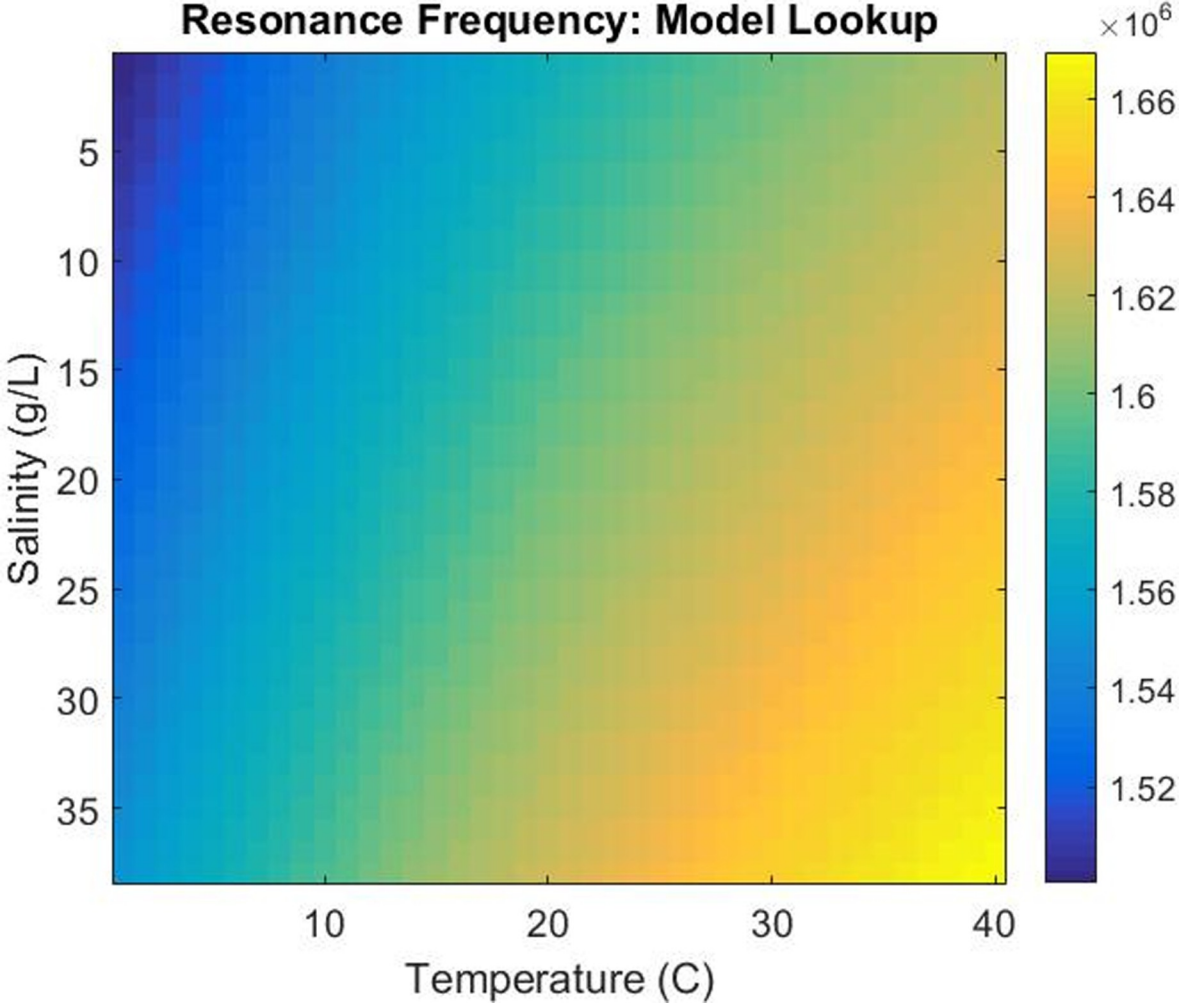
Resonance reference table. An environmental reference table for the capillary system’s resonance frequency (indicated by the color scale) modelled across a wide range of temperatures and salinities.

We suggest that an automated resonance control system that uses temperature, salinity, and conductance data will allow for the highest performance over the largest range of conditions. Such a system would utilize a simple two-part algorithm to 1) use the temperature and salinity characterization at a coarse level to limit the frequency sweep to a small ~10–20 kHz bandwidth (see Fig 11) followed by 2) an impedance/conductance scan as fine-tuning over this narrow bandwidth to find and maintain the local conductance max. This integrated approach would allow for a rapid resonance control methodology that can be operated in real time with very short windows of off-resonance drive during the rapid conductance scan.

This resonance control strategy has the potential to increase the performance of acoustic standing wave systems by selecting and maintaining the optimal driving frequency. As compared to standing wave systems that rely on highly controlled temperature and media, we have demonstrated a simple methodology that works across a wide range of conditions. Even systems that utilize highly controlled conditions will experience small resonance shifts and could benefit from the tight frequency control achieved in the local conductance scans. The ability to achieve consistent acoustic focusing over time will be integral to the development of acoustic focusing systems for a variety of application platforms. Specific examples include precise separation platforms (e.g. circulating tumor cells from blood), centrifuge-free washing of cells from sample media, as well as focusing for field-based interrogations such as electrical impedance spectroscopy or flow cytometry.

## Conclusions

Acoustic focusing systems have shown enormous promise for biological particle manipulations, yet their sensitivity to environmental shifts can lead to inconsistent performance. Here, we have described a comprehensive resonance control strategy to maintain performance across a wide range of environmental conditions and have demonstrated its viability within two discrete microfluidic device platforms. Our experimental platform, coupling a low-power frequency sweep and image-analysis of the particle positions, creates a relatively simple, yet powerful method of extracting the ideal operating frequency of an acoustic focusing device across any range of sample conditions. Using this methodology with a steel capillary device across a wide range of temperatures and salinities, we observe that the speed of sound within the liquid layer dominates the resonance frequency shift with temperature. Additionally, our impedance measurements show that there is a local conductance maximum associated with the best-focusing frequency for both the steel capillary and etched Si wafer systems. The conductance spectra are sufficiently complicated, exhibiting multiple resonance features, such that if the frequency search is not limited to a local area, one could easily drive a sub-optimal frequency. However, when coupled with prior knowledge extracted from the temperature scans, the frequency search can easily be constrained to the correct region. Operating at this local conductance maximum yields a simple, rapid, and highly accurate method of maintaining optimal system performance. This resonance control strategy greatly improves the consistency, performance, and commercial viability of any number of microfluidic acoustic standing wave systems.

## Supporting information

S1 Fig Custom cytometer for etched silicon device monitoring. The custom imaging-in-flow cytometry platform used to characterize the etched silica system.
(TIF)

S2 Fig Calibration of impedance data. A 10ohm resistor is measured on a calibrated Agilent impedance analyzer across the frequency range of interest (1–1.5 Mhz). This Agilent data on the resistor is then used as a ‘gold standard’ and the real and imaginary parts are used to calibrate our custom oscilloscope-based system to the Agilent data. A linear fit across the frequency space of interest is used to correct the oscilloscope data back to the Agilent data. The voltage going to the PZT is split and measured in parallel on a high impedance channel on the oscilloscope, while a current loop (Pearson) is used for the current measurement. The voltage and current signal are acquired into LabVIEW where the magnitude and phase information is extracted. Top: There is a small-negatively sloped resistance offset that is corrected to the standard value during the calibration. Middle: There is a large reactance offset between the calibrated and uncalibrated system that is corrected during the calibration. Bottom: Combining the real/imaginary components, the magnitude of the impedance is offset and non-linear unless it is correctly calibrated.
(TIF)

S3 Fig COMSOL model. A) A variety of eigenfrequencies around 1.5MHz are overlaid in the 1D Comsol model. The model system is Air/PZT/Steel/Water/Steel/Air. There are a lot of frequencies that exhibit high leakage out into the surrounding air, and only a few frequencies that exhibit a high pressure within the liquid sample. The best eigenfrequency corresponds to the half-wavelength mode where the pressure in the sample layer is maximized (Pink plot above). B) The best eigenfrequency for the COMSOL Model system. The air-backed PZT drives near its half-wavelength resonance mode, approximately a quarter wavelength goes through the steel layer while the liquid sample layer is driven at its fundamental half-wavelength resonance mode that optimizes performance. The model system is subjected to a variety of temperatures and the frequency where the energy is maximized within the liquid layer is selected at each temperature point.
(TIF)

S4 Fig Individual focusing scans for each temperature point. A) All temperature scan focusing data overlaid onto a single plot. The focusing minima where acoustic focusing performance is maximized are extracted to create plots of optimal focusing frequency vs. temperature. B) All temperature scan focusing data as individual plots. The focusing minima where acoustic focusing performance is maximized are extracted to create plots of optimal focusing frequency vs. temperature.
(TIF)

S5 Fig Raw focusing data for salinity scans in capillary. Focusing width in capillary as a function of frequency is plotted across a range of salinities.
(TIF)

S6 Fig Raw focusing data for salinity scans in etched silica system. A) The focusing width vs frequency data for all five salinity conditions with the silica system. There is essentially a binary shift in optimal frequency conditions. B) The optimal focusing frequency vs salinity for the silica system. Although the model does not capture a binary shift, it does catch the trend within a bandwidth ~15kHz. Such a coarse characterization will be sufficient when combined with a local conductance scan. C) Raw Salinity Data for etched silica system. Overlaid focusing and conductance data across salinities.
(TIF)

S7 Fig Q and bandwidth data. For the Si system, the bandwidths at half-maximum are extracted for both the focusing minima and the conductance maxima at the optimal resonance frequency (1.179MHz). The conductance scan has a sharp, relatively high Q and narrow bandwidth peak that can be used for precise control of the resonance frequency. The wider/flatter focusing minimum would make it slightly less selective for frequency control. Additionally, the focusing data requires a statistically significant number of particles to quantify the focusing width, and this takes time. In contrast, the conductive scan is purely electrical and can be conducted very rapidly.
(TIF)

S8 Fig High-power scan overlaid with the conductance spectra. Much like the low power scan, there is a clear conductance maximum at the single highest performing frequency (1.179 MHz). However, in addition to this one local condition, we see that the other local minima of high focusing performance are typically correlated with a local conductance maximum.
(TIF)
