# Supplementary figures and images for: Resonance control of acoustic focusing systems through an environmental reference table and impedance spectroscopy
Source: PLoS One. 2018 Nov 14;13(11):e0207532. doi: 10.1371/journal.pone.0207532 (PMC6235394; doi:10.1371/journal.pone.0207532)

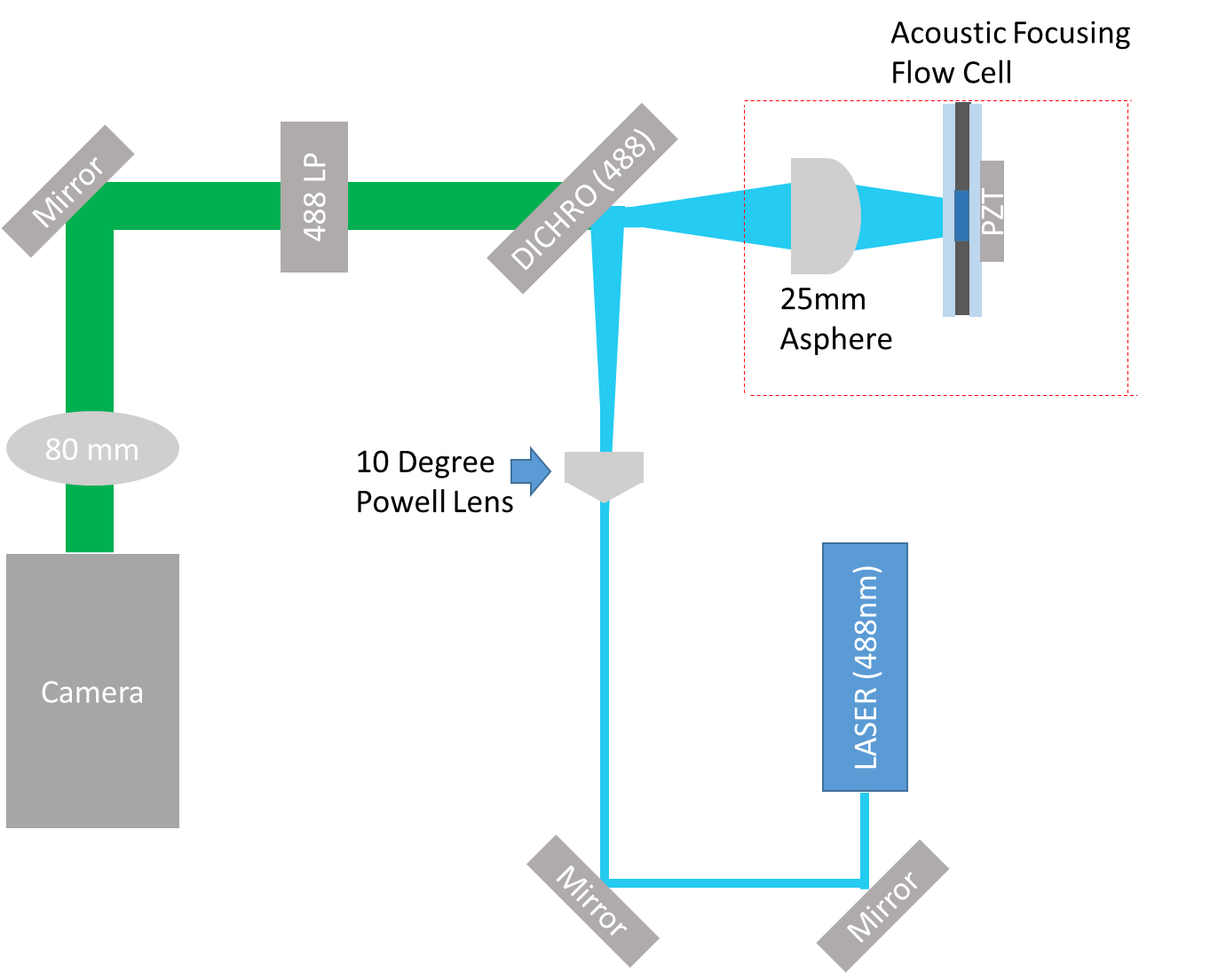

Supplement: S1 Fig — The custom imaging-in-flow cytometry platform used to characterize the etched silica system. (TIF) [file pone.0207532.s001.tif]

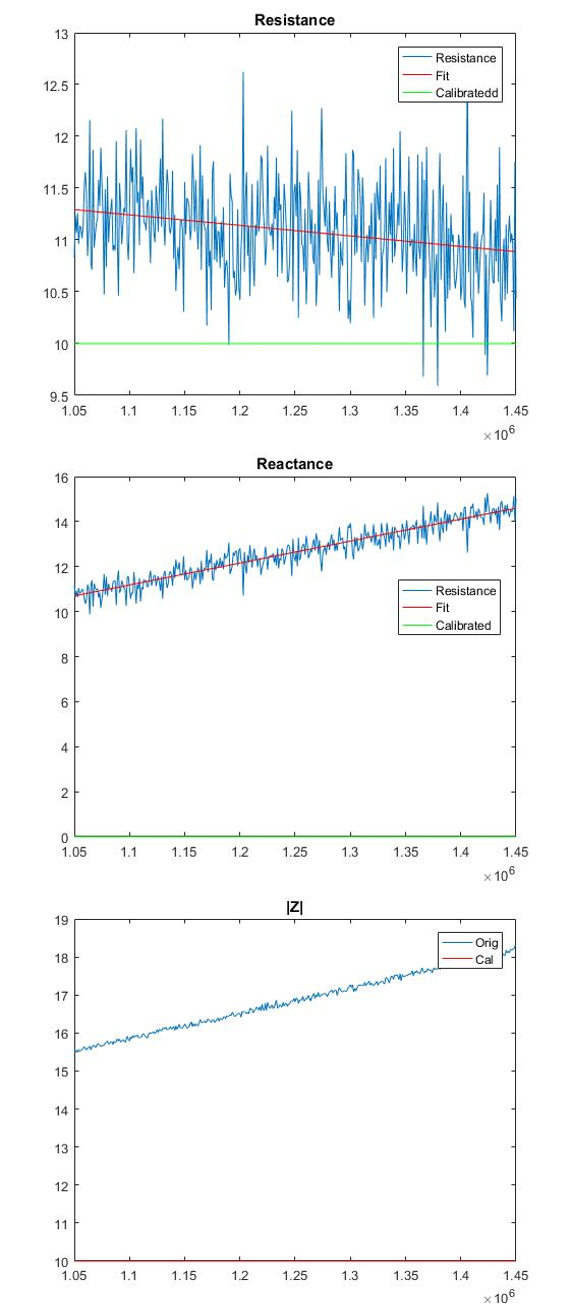

Supplement: S2 Fig — A 10ohm resistor is measured on a calibrated Agilent impedance analyzer across the frequency range of interest (1–1.5 Mhz). This Agilent data on the resistor is then used as a ‘gold standard’ and the real and imaginary parts are used to calibrate our custom oscilloscope-based system to the Agilent data. A linear fit across the frequency space of interest is used to correct the oscilloscope data back to the Agilent data. The voltage going to the PZT is split and measured in parallel on a high impedance channel on the oscilloscope, while a current loop (Pearson) is used for the current measurement. The voltage and current signal are acquired into LabVIEW where the magnitude and phase information is extracted. Top: There is a small-negatively sloped resistance offset that is corrected to the standard value during the calibration. Middle: There is a large reactance offset between the calibrated and uncalibrated system that is corrected during the calibration. Bottom: Combining the real/imaginary components, the magnitude of the impedance is offset and non-linear unless it is correctly calibrated. (TIF) [file pone.0207532.s002.tif]

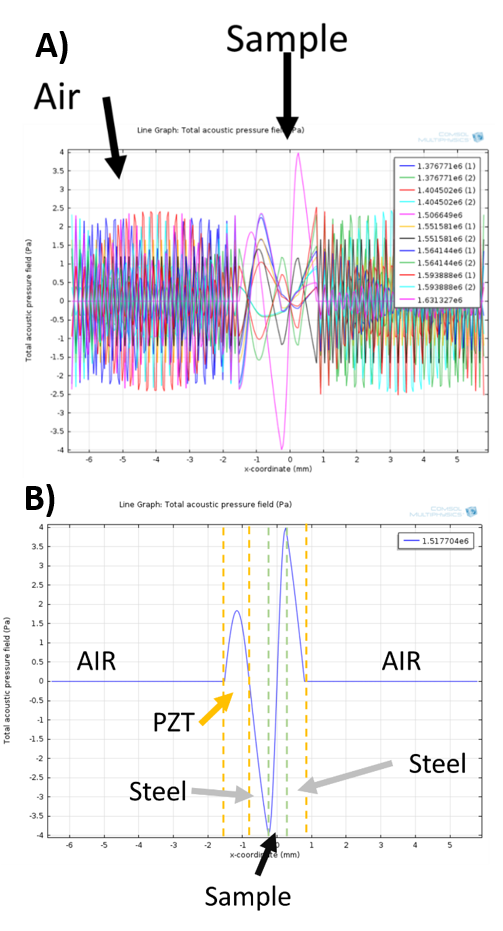

Supplement: S3 Fig — A) A variety of eigenfrequencies around 1.5MHz are overlaid in the 1D Comsol model. The model system is Air/PZT/Steel/Water/Steel/Air. There are a lot of frequencies that exhibit high leakage out into the surrounding air, and only a few frequencies that exhibit a high pressure within the liquid sample. The best eigenfrequency corresponds to the half-wavelength mode where the pressure in the sample layer is maximized (Pink plot above). B) The best eigenfrequency for the COMSOL Model system. The air-backed PZT drives near its half-wavelength resonance mode, approximately a quarter wavelength goes through the steel layer while the liquid sample layer is driven at its fundamental half-wavelength resonance mode that optimizes performance. The model system is subjected to a variety of temperatures and the frequency where the energy is maximized within the liquid layer is selected at each temperature point. (TIF) [file pone.0207532.s003.tif]

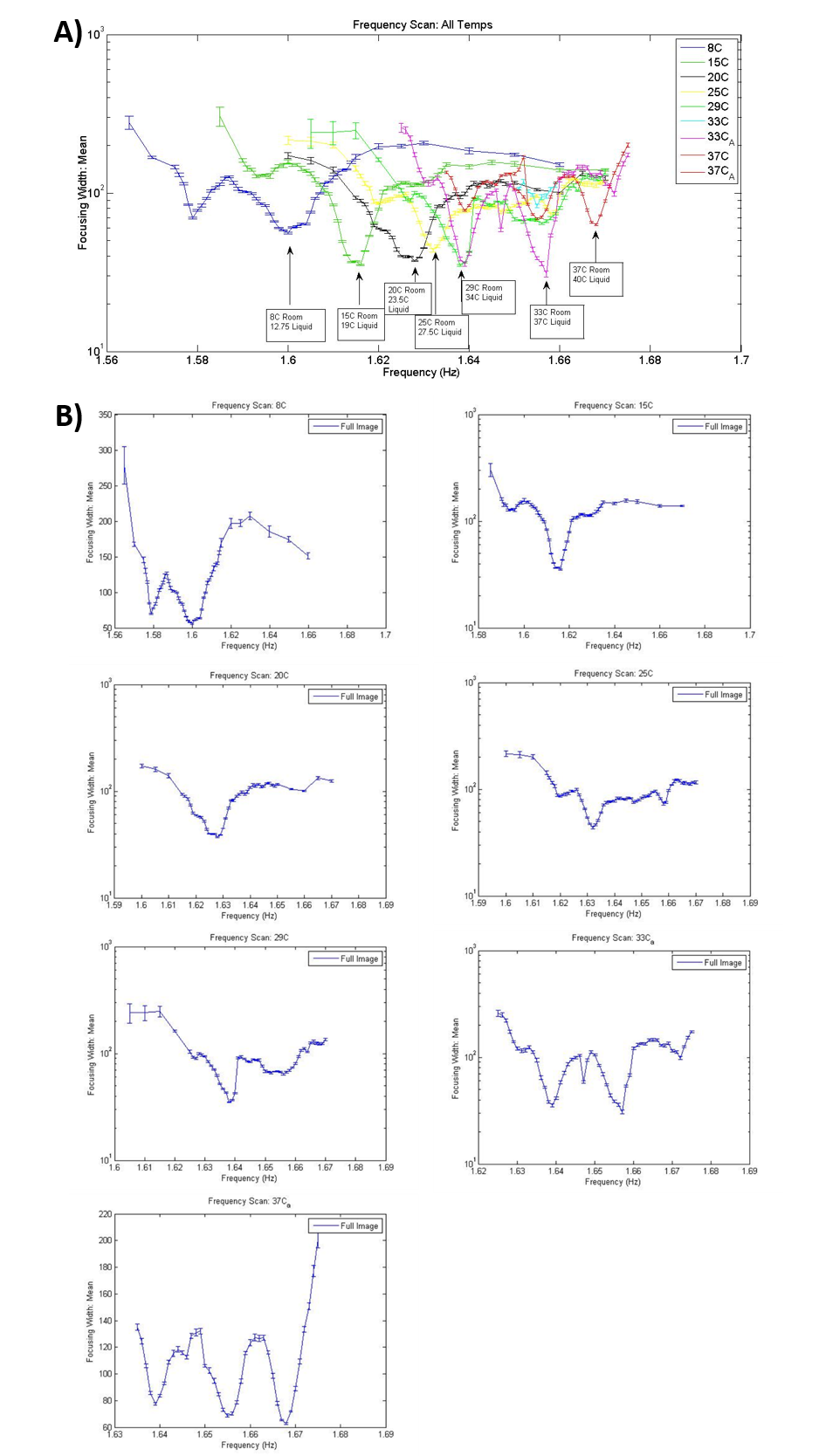

Supplement: S4 Fig — A) All temperature scan focusing data overlaid onto a single plot. The focusing minima where acoustic focusing performance is maximized are extracted to create plots of optimal focusing frequency vs. temperature. B) All temperature scan focusing data as individual plots. The focusing minima where acoustic focusing performance is maximized are extracted to create plots of optimal focusing frequency vs. temperature. (TIF) [file pone.0207532.s004.tif]

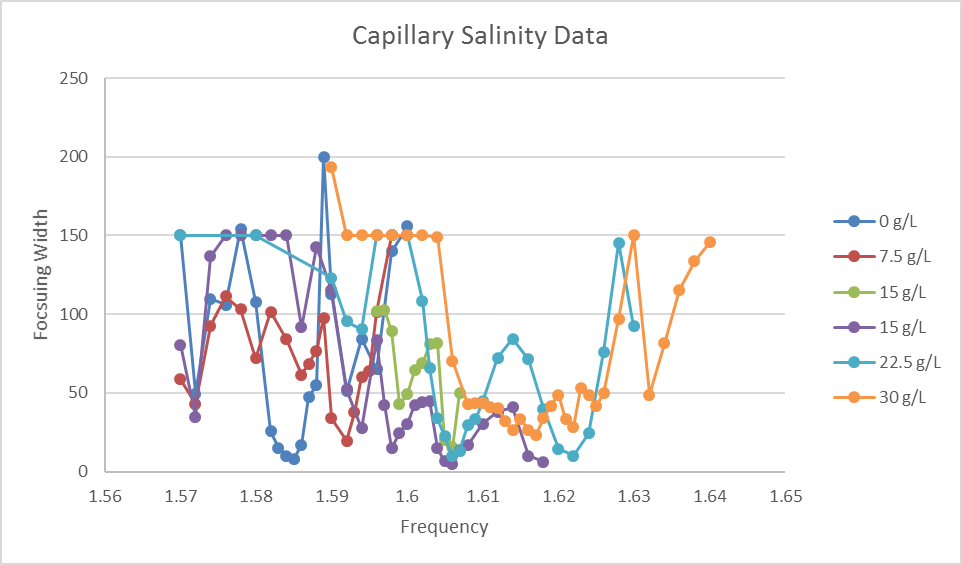

Supplement: S5 Fig — Focusing width in capillary as a function of frequency is plotted across a range of salinities. (TIF) [file pone.0207532.s005.tif]

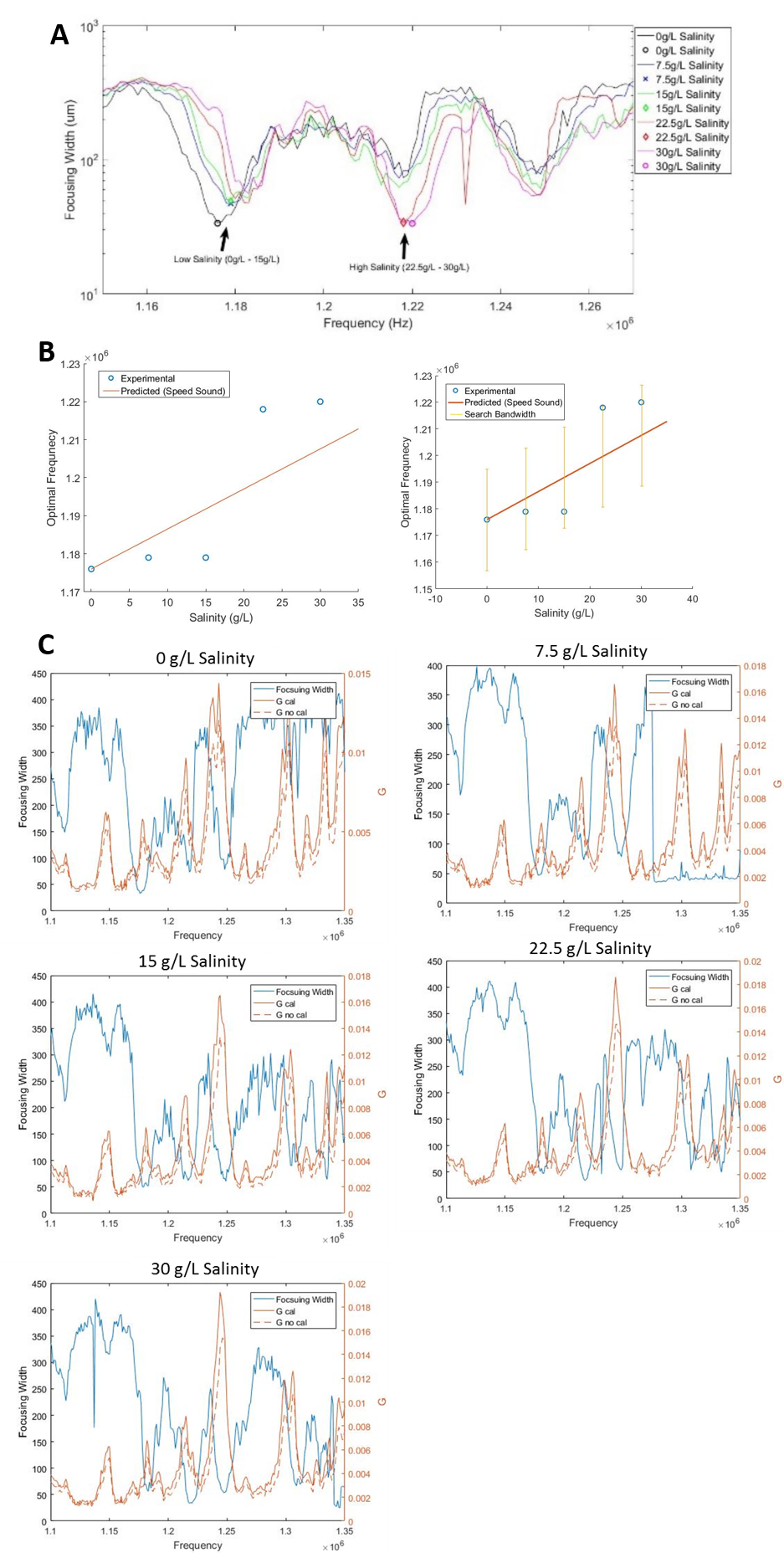

Supplement: S6 Fig — A) The focusing width vs frequency data for all five salinity conditions with the silica system. There is essentially a binary shift in optimal frequency conditions. B) The optimal focusing frequency vs salinity for the silica system. Although the model does not capture a binary shift, it does catch the trend within a bandwidth ~15kHz. Such a coarse characterization will be sufficient when combined with a local conductance scan. C) Raw Salinity Data for etched silica system. Overlaid focusing and conductance data across salinities. (TIF) [file pone.0207532.s006.tif]

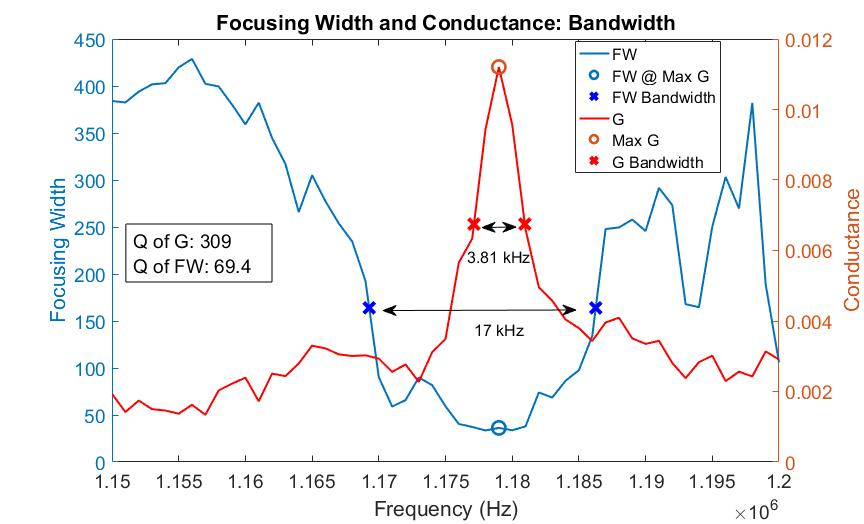

Supplement: S7 Fig — For the Si system, the bandwidths at half-maximum are extracted for both the focusing minima and the conductance maxima at the optimal resonance frequency (1.179MHz). The conductance scan has a sharp, relatively high Q and narrow bandwidth peak that can be used for precise control of the resonance frequency. The wider/flatter focusing minimum would make it slightly less selective for frequency control. Additionally, the focusing data requires a statistically significant number of particles to quantify the focusing width, and this takes time. In contrast, the conductive scan is purely electrical and can be conducted very rapidly. (TIF) [file pone.0207532.s007.tif]

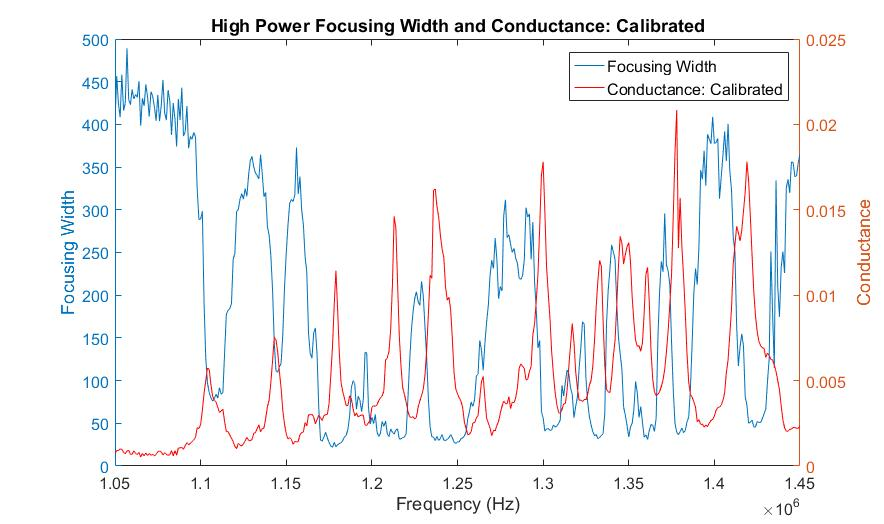

Supplement: S8 Fig — Much like the low power scan, there is a clear conductance maximum at the single highest performing frequency (1.179 MHz). However, in addition to this one local condition, we see that the other local minima of high focusing performance are typically correlated with a local conductance maximum. (TIF) [file pone.0207532.s008.tif]
